# Aboveground competition influences density‐dependent effects of cordgrass on sediment biogeochemistry

**DOI:** 10.1002/ece3.8722

**Published:** 2022-03-22

**Authors:** Janet B. Walker, Shelby Rinehart, Gabriel Greenberg‐Pines, Wendi K. White, Ric DeSantiago, David A. Lipson, Jeremy D. Long

**Affiliations:** ^1^ 7117 Department of Biology San Diego State University San Diego California USA; ^2^ 7117 Coastal and Marine Institute San Diego State University San Diego California USA; ^3^ 271871 Southern California Coastal Water Research Project Costa Mesa California USA; ^4^ Department of Biological Sciences University of Alabama Tuscaloosa Alabama USA; ^5^ Department of Zoology and Biodiversity Research Centre University of British Columbia Vancouver BC Canada; ^6^ 14708 Department of Biology University of Massachusetts Boston Boston Massachusetts USA; ^7^ 8789 Department of Environmental Science and Policy University of California Davis Davis California USA

**Keywords:** ecosystem function, interspecific competition, nitrogen cycling, plant–plant interactions, plant–soil feedbacks, salt marshes

## Abstract

Interspecific interactions between plants influence plant phenotype, distribution, abundance, and community structure. Each of these can, in turn, impact sediment biogeochemistry. Although the population and community level impacts of these interactions have been extensively studied, less is known about their effect on sediment biogeochemistry. This is surprising given that many plants are categorized as foundation species that exert strong control on community structure. In southern California salt marshes, we used clipping experiments to manipulate aboveground neighbor presence to study interactions between two dominant plants, Pacific cordgrass (*Spartina foliosa*) and perennial pickleweed (*Sarcocornia pacifica*). We also measured how changes in cordgrass stem density influenced sediment biogeochemistry. Pickleweed suppressed cordgrass stem density but had no effect on aboveground biomass. For every cordgrass stem lost per square meter, porewater ammonium increased 0.3–1.0 µM. Thus, aboveground competition with pickleweed weakened the effects of cordgrass on sediment biogeochemistry. Predictions about plant–soil feedbacks, especially under future climate scenarios, will be improved when plant–plant interactions are considered, particularly those containing dominant and foundation species.

## Graphical abstract

Plant interspecific interactions are well‐documented across ecosystems and play a critical role in determining plant phenotypes, abundance, and distribution. However, despite the known feedbacks between plants and soil, there have been few attempts to link plant interspecific interactions to changes in soil chemical properties. Our work bridges this gap using a historical model system in tidal wetlands. We found that aboveground competition between cordgrass and pickleweed reduced the stem density of cordgrass which, in turn, increased the ammonium concentrations in sub‐surface marsh sediments.

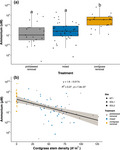

## 1 INTRODUCTION

Interspecific interactions affect the population dynamics of plants (Bertness & Callaway, 1994; Brooker et al., 2008; Gornall et al., 2011). These population‐level effects may have important consequences for ecosystem function since plant populations often influence local sediment conditions (e.g., nitrogen‐fixing plants can alter local sediment biogeochemistry; Vitousek & Walker, 1989, Haubensak & Parker, 2004). Despite our understanding of plant–plant competition and the ecosystem‐level impacts of plant populations, ecologists have made few attempts to link competition between plants with local ecosystem functions such as biogeochemical cycling (but see Box 2 in De Long et al., 2019). Consequently, plant–plant interactions may not initially be included in models of plant‐soil feedbacks (see Figure 2 in De Long et al., 2019). As plant communities continue to change with range shifts linked to climate change and species introductions, there is a pressing need to better understand the connection between plant competition and soil biogeochemistry.

Interspecific interactions modify the distribution and abundance of plants (see reviews by Connell, 1983; Goldberg & Barton, 1992; Gurevitch et al., 1992; Harper, 1977; Schoener, 1983). For example, interspecific competition restricts the distribution of grasses in ridge crests (Gurevitch, 1986), salt marshes (Guo & Pennings, 2012), and alpine meadows (Theodose & Bowman, 1997). The consequences of such competition‐mediated shifts in plant communities may be especially profound when one of the interacting species plays an essential role in their local community (e.g., foundation species).

Competition‐mediated changes in plant traits and communities could modify important ecosystem functions via species‐specific effects on below‐ground processes (Kelly et al., 1998; Weidenhamer & Callaway, 2010). Such species‐specific effects of plants can cause important changes to the soil environment, hydrology, climate, and biogeochemical cycling. As noted by Eviner and Chapin (2003), “Plant species can differ in their effects on almost every aspect of ecosystem structure and function.” For example, grasses (1) uptake water more efficiently than forbs (Gordon et al., 1989; Güsewell, 2004), (2) reduce evapotranspiration relative to deep‐rooted trees (Shukla et al., 1990), (3) provide better aggregate stability than other plant groups (Jastrow, 1987; Wright & Anderson, 2000), and (4) stabilize soil silica via production of phytoliths (Kelly et al., 1998; Song et al., 2012). Such species‐specific effects provide a mechanism by which plant–plant competition could commonly influence ecosystems—yet few studies have established this relationship.

In salt marsh ecosystems, cordgrass (*Spartina* spp.) is a foundation species that provides critical habitat for animals (Boyer & Zedler, 1996, 1998; Gratton & Denno, 2005), stabilizes marsh sediments (Meyer et al., 1997), and modifies sediment biogeochemistry (Koop‐Jakobsen & Giblin, 2009). The abundance and distribution of cordgrass are commonly affected by interspecific interactions with neighboring marsh plants. For example, other plant species limit the upper distribution of *Spartina* spp. in northeastern and southeastern US salt marshes (Bertness & Ellison, 1987; Pennings et al., 2005; respectively). Similarly, pickleweed reduces cordgrass productivity in southern California (i.e., *S*. *foliosa*; Covin & Zedler, 1988, Boyer & Zedler, 1999).

Such competitive interactions could influence marsh sediment biogeochemistry. Unlike co‐occurring plants, cordgrass can oxygenate subsurface sediments via specialized below‐ground tissues called aerenchyma (Howes & Teal, 1994). By oxygenating sediments, cordgrass creates microclimates that promote the uptake of limiting nutrients, like ammonium (Morris & Dacey, 1984). Plants that suppress cordgrass growth should increase nutrient availability, at least at subsurface sediment depths. Although (1) cordgrass is commonly outcompeted by upper marsh species and (2) cordgrass shapes sediment biogeochemistry, we lack an understanding about how competition mediates the impact cordgrass has on edaphic conditions. Evaluating the links between plant–plant competition and ecosystem functions in marshes is especially critical and timely, as anthropogenic climate change is known to affect the competitive abilities of dominant plant species, like pickleweed (Noto & Shurin, 2017).

Here, we assess the link between plant interspecific interactions and salt marsh ecosystem function across multiple sites in southern California via clipping manipulations of plants in the field. We focused on the interaction between two dominant salt marsh plants, Pacific cordgrass (*Spartina foliosa*) and perennial pickleweed (*Sarcocornia pacifica*). We used this model system to understand the impact of plant–plant interactions on sediment biogeochemistry. We predicted that interactions with pickleweed would suppress cordgrass and thereby weaken the effects of cordgrass on sediment biogeochemistry, measured as porewater chemistry and iron fractionation (Bertness & Ellison, 1987; Covin & Zedler, 1988). Specifically, dominant species that suppress cordgrass growth should enhance sediment ammonium.

## 2 METHODS

### 2.1 Study sites and species

To understand neighbor effects on Pacific cordgrass populations and the indirect effects of neighbors on local sediment biogeochemistry, we conducted a fully factorial experiment in a salt marsh transition zone, manipulating cordgrass (*Spartina foliosa*) and pickleweed (*Sarcocornia pacifica*) stem density. This created three types of plots: Mixed plots (containing intact and unmanipulated cordgrass and pickleweed), Cordgrass Removal plots, and Pickleweed Removal plots. We deployed this experiment at three sites: two sites were in San Dieguito Lagoon (SDL1: 32°58′47.0″N, 117°14′43.6″ W; SDL2: 32°58′44.2″N 117°14′39.6″W; Del Mar, CA) and one site was in Kendall‐Frost Marsh (KF1: 32°47′39.8″N 117°13′46.6″ W; San Diego, CA). The study was conducted in 2016 (KF1 and SDL1) and 2017 (SDL2). At each site, we installed plots at intermediate elevations dominated by a mixture of cordgrass and pickleweed. Subordinate plants were more common at Kendall‐Frost Marsh, and included *Jaumea carnosa*, *Salicornia bigelovii*, and *Batis maritima*. Subordinate plants may be less common at San Dieguito Lagoon because it is an active restoration site, where only *S*. *foliosa* and *S*. *pacifica* were transplanted in 2009 and 2011. Kendall‐Frost Marsh consists of about 40 acres of natural marsh that once spanned more than half of Mission Bay (San Diego, CA), prior to its transformation in the late 1940s (Levin, 1984; Moseman et al., 2009). The difference in marsh age between the sites could affect many ecosystem properties, such as landscape characteristics, hydrological modification, and biologic variables (vegetation cover, benthic infauna abundance, etc.; Staszak & Armitage, 2013).

### 2.2 Experimental manipulation

At all sites, we haphazardly selected 0.5 × 0.5 m plots in the transition zone, and then selected plots that contained both cordgrass and pickleweed and standardized percent cover for each plant between 40% and 60%. Plots were marked by placing 75 cm PVC pipes at two, diagonal corners. We randomly assigned plots to one of three treatments: Cordgrass Removal, Pickleweed Removal, and Mixed (*n* = 7–10 per treatment, Appendix S1: Table S1). Treatments were created by clipping neighbor species (i.e., pickleweed in Pickleweed Removal plots and cordgrass in Cordgrass Removal plots) at the soil surface. Our clipping approach is commonly employed to study plant–plant interactions, especially in salt marshes (e.g., Bertness & Ellison, 1987; Boyer & Zedler, 1999; Covin & Zedler, 1988). We maintained these treatments by clipping removed plants every 2–3 weeks throughout the growing season for both plants (April–September). We did not clip any plants in Mixed plots. Clipping neighboring species should alleviate aboveground interspecific interactions, while having weaker effects on belowground interactions, since plant rhizomes remain intact.

### 2.3 Plant characteristics

To assess plant responses to neighbor removals, we nondestructively sampled several plant and community characteristics (e.g., cordgrass plant height, cordgrass stem density, pickleweed canopy height, and plant cover). Cordgrass plant height was measured by haphazardly selecting 10 cordgrass plants and measuring plant height from the soil–plant interface to the apical tip. Stem density was calculated by dividing the number of stems in plots by plot area. We measured pickleweed canopy height as the distance from the soil–plant interface to the tallest peak of the pickleweed canopy. We assessed the percent cover of the plant canopy nondestructively by placing a quadrat (0.5 × 0.5 m) on each plot and recording the uppermost species or substrate beneath 100 evenly spaced sampling nodes (4.5 cm apart). We measured plant traits about every 2 months throughout the growing season, however, we only report data from the last sampling month (September).

At the end of the growing season, we harvested the aboveground biomass in each plot by clipping all plants at the plant–soil interface. Harvested plants were sorted by species (i.e., cordgrass, pickleweed, and other less common species) and dried at 60°C for 4 days before a final dry biomass per plot was obtained. Additionally, at SDL2 only, we extracted 27‐cm‐deep sediment cores (Volume ~3,980 cm^3^) from the middle of each plot. These samples were transported to San Diego State University's Coastal and Marine Institute Laboratory (San Diego, CA), where we sieved sediment cores through 1 mm mesh to remove belowground roots. Root material was then identified to species (cordgrass or pickleweed), under a compound microscope where necessary, and placed in the drying oven at 60°C for 4 days before the final dry mass was obtained. Roots too small to identify to species were classified as “unknown.” Belowground biomass was extrapolated across the entire plot (0.5 × 0.5 × 0.27 m, length × width × depth, 0.0675 m^3^).

### 2.4 Biogeochemistry

To understand how neighbor manipulations affected sediment properties, we monitored sediment biogeochemistry throughout the study. We installed porewater samplers [porous (0.15 µm) soil moisture samplers; Rhizophere Research Products, Wageningen, Netherlands] at each site by inserting sippers in plot centers and perpendicular to the mud surface at a constant depth (10 cm; Appendix S1: Table S1). Most of the cordgrass rhizome occurs between 10 and 20 cm soil depth (Hackney & de la Cruz, 1986). Porewater samples were collected about every 2 months during low tide, and frozen at −80℃ until analyzed.

From these porewater samples, we analyzed salinity, dissolved organic carbon (DOC), nitrate, and ammonium concentrations (*sensu* Lipson et al., 2012). Salinity was measured with a refractometer. DOC, nitrate, and ammonium were measured with colorimetric assays (SpectraMax 190, Molecular Devices, San Jose, California, USA). DOC was measured using an index of dissolved aromatic compounds and absorbance was recorded at 260 nm (A260) using a UV‐transparent microtiter. Nitrate was measured using vanadium III, Griess reagents with standards made from artificial seawater, and absorbance was recorded at 540 nm (Miranda et al., 2001). Ammonium was measured using a phenolate–hypochlorate chemical analysis, standards of artificial seawater, and absorbance was recorded at 650 nm (U.S. EPA, 1983).

At one of our sites (SDL2), we conducted soil iron fractionation as a proxy for sediment oxygen (*sensu* Lipson et al., 2010). We only quantified Fe (III) at SDL2 because of logistical constraints. We quantified the redox state of acid‐extractable Fe because Fe (III) provides evidence of oxygenated sediment conditions. We collected a single 5 cm diameter sediment core from the center of a randomly selected subset of plots (*n* = 4 for Pickleweed and Cordgrass Removal plots, *n* = 8 for Mixed plots; Appendix S1: Table S1). Because cordgrass rhizomes tend to be deeper than pickleweed roots, we partitioned sediment cores into two depths, 1–10 cm and 10–20 cm. Upon collection, samples were placed into 50 ml polypropylene tubes with 20 ml of 1 M HCl. Samples were then transported to San Diego State University and weighed in the lab before being shaken overnight at 120 rpm. Samples were then centrifuged and analyzed using 1, 10‐ *o* ‐ phenanthroline, which undergoes a reaction with Fe (II) (Lipson et al., 2010). Ascorbic acid was added to determine the total soluble Fe. Fe (III) concentrations were calculated as the difference between total soluble Fe and Fe (II) (Knorr & Blodau, 2009; Lipson et al., 2010; Tamura et al., 1974). Assays were completed on a spectrophotometer (SpectraMax 190, Molecular Devices, San Jose, California, USA). We report the proportion of Fe (III) in the total soluble Fe pool.

### 2.5 Data analysis

To assess the effects of plant communities on plant and porewater metrics, we used Linear Mixed Effect Models (LMEMs) and Generalized Linear Mixed Effects Models (GLMMs) due to their ability to accommodate both non‐normal distributions and heterogeneity in variances (Bolker et al., 2008; Schielzeth et al., 2020; Venables & Dichmont, 2004). For all models, except salinity and DOC, we included treatment as a fixed factor, site as a random effect, and initial cordgrass stem density as a covariate. We were most interested in the effects of neighbors and the effects of competition on biogeochemistry across all sites, rather than the effects at individual sites. By including site as a random effect, we were able to account for the natural variation among sites in southern California salt marshes. Additionally, by including initial cordgrass stem density as a covariate, we accounted for the potential legacy effects caused by initial cordgrass stem density before treatments were assigned. Post‐hoc tests were carried out with Tukey's HSD test (*α* = .05).

To assess the effect of plant communities on cordgrass plant height, cordgrass stem density, and pickleweed canopy height, we used LMEMs (after log transforming when necessary). We only included treatments that contained the focal plant (i.e., Pickleweed Removal and Mixed plots for cordgrass height and stem density, Cordgrass Removal and Mixed plots for pickleweed canopy height). To understand the effects of treatment on plant cover, we ran a GLMM examining the effect of neighbors on the sum of cordgrass and pickleweed cover. Due to the exponentially distributed data, we used a log link function and a dispersion parameter set to 1.

To understand how plant communities affect aboveground biomass, we ran a single GLMM with treatment as a fixed factor, plant (cordgrass or pickleweed) as a fixed factor, the interaction of the fixed factors (treatment and plant), initial cordgrass stem density as a covariate, and site as a random effect. Due to the clipping of neighbors in our treatments, cordgrass removal treatments and pickleweed removal treatments had zero cordgrass and pickleweed aboveground biomass, respectively. To account for this in our models, we added a zero‐inflation parameter (using the R package, glmmTMB; Brooks et al., 2017). Unlike aboveground biomass, we did not manipulate belowground biomass, thus removal plots had both cordgrass and pickleweed roots. Therefore, we conducted a log transformation and ran a single linear model with treatment as a fixed factor, plant (cordgrass or pickleweed) as a fixed factor, the interaction of treatment and plant, and initial cordgrass stem density as a covariate.

To examine neighbor effects on sediment biogeochemistry, we log transformed ammonium and nitrate and ran separate LMEMs for these nutrients. Because salinity and DOC were bimodally distributed between sites, we ran separate GLMs for each site. We assessed the proportion of Fe (III) in the total Fe pool at SDL2 by running a full linear model with treatment and depth (0–10 cm and 10–20 cm) as fixed factors, the interaction of treatment and depth, and initial cordgrass stem density as a covariate. For all sediment biogeochemistry, we dropped samples that were non‐detects.

When we examined treatment as a categorical independent variable, we did not observe an effect of treatment on porewater ammonium (or proportional Fe(III)). However, there was considerable within‐treatment variation in ammonium at each site (Coefficient of Variation = .836, 1.093, and 1.277, for Cordgrass Removal, Mixed, and Pickleweed Removal treatments, respectively). Because stem density is strongly linked to ammonium levels (and Fe (III); Mozdzer et al., 2011), we suspected that large within‐treatment variation in final stem density (see above) impaired our ability to detect an effect of neighbors on ammonium (i.e., a treatment effect). To explore the relationship between cordgrass stem density and sediment metrics, we used linear regressions with final cordgrass stem density as the independent variable and either ammonium or proportional Fe (III) as the response variable. For ammonium, we ran a LMEM with cordgrass stem density and site as random effects. For proportional Fe (III) (only measured at SDL2), we ran separate regressions for each depth due to the importance of depth (as found in the full model).

Statistical analyses were performed using R software v. 4.0.2 (R‐Core‐Team, 2020). Analyses were conducted in R using the lme4 package for LMEMs and GLMMs (Bates et al., 2015) and glmmTMB package for zero‐inflation mixed effect models (Brooks et al., 2017). We tested significance of fixed effects with type II sums of squares using the *Anova* function in the car package (Fox & Weisberg, 2019).

## 3 RESULTS

### 3.1 Plant characteristics

**FIGURE 1:**
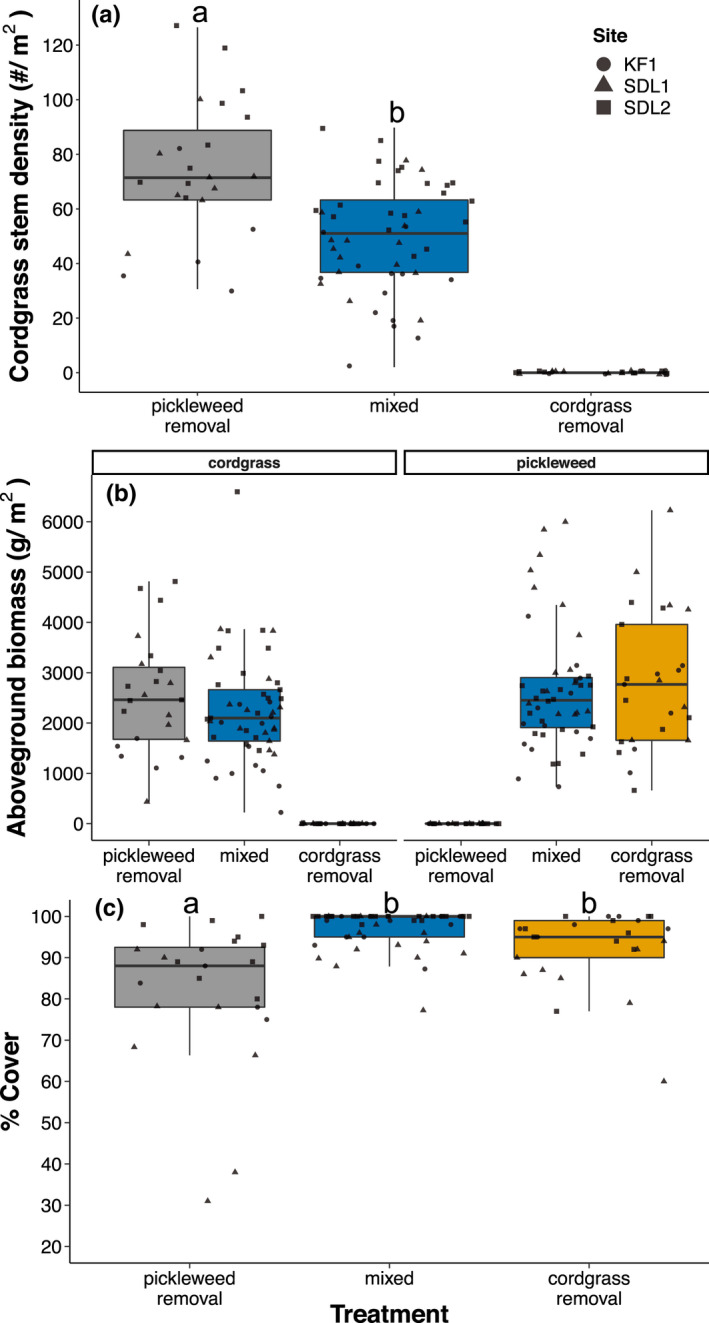
(a) Cordgrass stem density, (b) aboveground biomass for each plant species (cordgrass and pickleweed), and (c) total cordgrass and pickleweed cover for each treatment. Lines inside boxes are median values, box limits are Q1 and Q3, and whiskers represent non‐outlier ranges. Letters represent significant differences between treatments (Tukey HSD test; *α* = .05). Zero values in Cordgrass Removal and Pickleweed Removal treatments reflect that cordgrass and pickleweed, respectively, were successfully manipulated in these treatments. Colors represent treatments and shapes represent sites

At each site, starting cordgrass stem densities were similar across treatments (KF1: *F* = .696, df = 2, *p* = .501; SDL1: *F* = .013, df = 2, *p* = .987; SDL2: *F* = .343, df = 2, *p* = .712; Appendix S1: Figure S1). Across all sites, removing pickleweed neighbors increased cordgrass stem density by 40% (Pickleweed Removal vs. Mixed plots; Table 1, Figure 1a). We also observed a trend for higher final stem density at sites with high initial stem density (e.g., stem densities were higher at SDL). There was no effect of neighbor removal on cordgrass stem height (Table 1, Appendix S1: Figure S2). Neighbor removals did not affect aboveground or belowground biomass (Figure 1b and Appendix S1: Figure S3, respectively; Appendix S1: Table S2).

**TABLE 1:** Output table of models of plant characteristics

	Dependent Variables											
	Cordgrass stem density			Cordgrass plant height			Pickleweed canopy height			Total cordgrass and pickleweed cover		
	*df*	χ^2^	*p*	*df*	χ^2^	*p*	*df*	χ^2^	*p*	*df*	χ^2^	*p*
Treatment	1	34.979	<.001	1	1.247	.264	1	5.464	.0194	2	32.514	<.001
Initial cordgrass stem density	1	21.11	<.001	1	3.727	.0535	1	0.765	.382	1	3.80	.051

Note

Results of models testing the effect of treatment (Cordgrass Removal, Pickleweed Removal, and Mixed) on cordgrass stem density, cordgrass plant height, pickleweed canopy height and total cordgrass and pickleweed cover. For cordgrass stem density, cordgrass plant height, and c.

Pickleweed canopy height was higher in Mixed plots than Cordgrass Removal plots (Table 1, Appendix S1: Figure S4). Similar to Pickleweed Removal plots, removing cordgrass did not affect pickleweed aboveground or belowground biomass (Figure 1b and Appendix S1: Figure S3, respectively; Appendix S1: Table S2). Mixed and Cordgrass Removal plots had greater total percent plant cover than Pickleweed Removal plots (Table 1, Figure 1c). Our results suggest that removing cordgrass did not affect plant cover or pickleweed biomass, but that it reduced pickleweed height. We hypothesize that cordgrass provided structure that allowed the same amount of pickleweed to extend further above the soil surface.

### 3.2 Biogeochemistry

**FIGURE 2:**
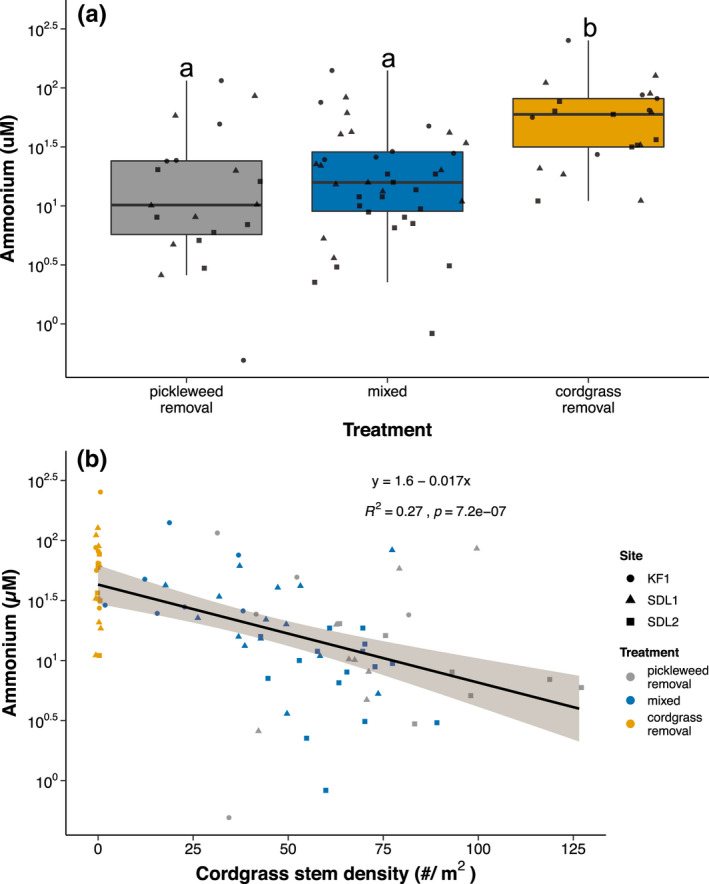
(a) Porewater ammonium concentrations for each treatment. Lines inside boxes are median values, box limits are Q1 and Q3, and whiskers represent non‐outlier ranges. Shapes represent site. Letters represent significant differences between treatments (Tukey HSD test; *α* = .05). (b) Porewater ammonium concentrations versus cordgrass stem density. Colors represent treatments and shapes represent sites

Ammonium concentrations differed between treatments and were highest in Cordgrass Removal plots (Figure 2a, Appendix S1: Table S3). Post‐hoc analyses revealed a significant difference between Cordgrass Removal and unmanipulated (Mixed) plots (*p* < .001). Removing cordgrass elevated ammonium levels by 60–75% compared to the other two treatments where cordgrass was unmanipulated. When we regressed ammonium with final cordgrass stem density, neighbor removal mediated increases in cordgrass stem density decreased ammonium concentrations (LMEM: *χ* ^2^ = 22.86, df = 1, *p* < .001; Figure 2b). For every cordgrass stem lost per square meter, ammonium increased by 0.3–1.0 µM. To determine if Removal plots were driving the relationship between stem density and ammonium, we conducted a separate regression with only Mixed plots. By including only Mixed plots, we tested whether natural variation in cordgrass stem density affected ammonium concentrations. Focusing only on these Mixed plots, cordgrass stem density and ammonium were negatively correlated (*F* = 14.369, df = 1, *p* < .001; Appendix S1: S5).

For all sites, there was no effect of neighbor removal on salinity or DOC (measured in UV absorbance; Appendix S1: Table S3, Figures S6 and S7). There was, however, an effect of neighbor removal on nitrate, where mixed treatments had a higher nitrate concentration than the other two treatments (Appendix S1: Table S3, Figure S8).

Treatment interacted with sediment depth to influence proportional Fe (III) [Appendix S1: Figure S9A; GLM (Gaussian), Interaction: *χ* ^2^ = 9.134, *df* = 2, *p* = .010]. At depths of 10–20 cm, the proportion of Fe (III) in Pickleweed Removal plots was 53% and 144% higher than in Mixed and Cordgrass Removal plots, respectively (Appendix S1: Figure S9). In contrast, in shallow sediments, there were no significant difference in treatments, but there was a trend for Pickleweed Removal plots to have the lowest proportions of Fe (III) in the total Fe pool. Proportional Fe (III) was only assessed at SDL2 as a proxy for sediment oxygenation. When we examined the relationship between stem density and Fe (III), neighbor‐removal‐mediated increases in cordgrass stem density increased Fe (III) in sub‐surface (10–20 cm), but not surface, sediments (Appendix S1: Figure S9B).

## 4 DISCUSSION

Aboveground interactions with pickleweed suppressed cordgrass stem density at transitional zones in southern California salt marshes. This competition was asymmetric—we saw no evidence of cordgrass suppressing pickleweed growth. Competition‐mediated reductions in cordgrass stem density were associated with increased sediment ammonium in sub‐surface sediments. This suggests that declines in cordgrass stems leads to reduced soil conditions with high concentrations of ammonium. Such changes in edaphic conditions are likely to affect the structure and functioning of salt marsh sediments and vegetation (Avrahami et al., 2002).

A paradigm in salt marsh ecology is that interactions with upper elevational plants suppress cordgrass species (Bertness et al., 1992; Chapman, 1974; Pennings et al., 2001, 2005). For example, saltwort (*Salicornia bigelovii*) reduced *Spartina foliosa* stem density (Boyer & Zedler, 1999) and upper elevation rush (*Juncus* spp.) decreased the aboveground production of *Spartina alterniflora* (Pennings et al., 2005) and *Spartina patens* (Bertness, 1991). Because pickleweed extends into higher elevations than cordgrass in Mediterranean salt marshes, our finding that pickleweed (*Sarcocornia pacifica*) suppressed cordgrass (*S*. *foliosa*) stem density provides support of this paradigm. Furthermore, stem density of cordgrass is commonly suppressed by these neighbors. For instance, saltworts (*Sarcocornia* sp.) suppressed stem density of cordgrass in Georgia (*S*. *alterniflora*; Angelini & Silliman, 2012) and southern California (*S*. *foliosa*; Boyer & Zedler, 1999).

Studies documenting competition‐mediated declines in cordgrass stem density often report comparable declines in cordgrass aboveground biomass. However, we saw no effects of competition on cordgrass aboveground biomass. While we did not directly measure the biomass per cordgrass stem, our finding suggests that cordgrass in our system alters its growth patterns in response to competition—producing higher numbers of stems with lower biomass per stem. Our study is not the first to observe altered growth patterns in cordgrass grown with competitors. Zerebecki et al. (2017) found that multiple cordgrass genotypes respond to neighbors by altering their growth patterns, but not their overall aboveground productivity. Together, our studies highlight the importance of quantifying multiple cordgrass traits when evaluating the impacts of competition on cordgrass productivity.

While competition with pickleweed reduced cordgrass stem density, final cordgrass stem density was also influenced by site‐specific differences in initial stem density. This pattern resulted largely from higher initial and final stem densities at San Dieguito Lagoon versus Kendall‐Frost Marsh. We observed no difference in starting stem density between treatments at any given site. Legacy effects of initial stem density are not surprising given the rhizomatous growth of cordgrass. Thus, predicting the outcomes of cordgrass interactions with neighboring plants may require a thorough understanding of starting conditions and their impact on the outcomes of plant–plant interactions.

Reductions in cordgrass stem density increased ammonium concentrations in sub‐surface (~10 cm depth) sediments. In addition to competition‐mediated changes in cordgrass stem density driving this pattern, it is possible that manipulations impacted sediments via changes in total plant cover. Our clipping manipulations reduced total plant cover, which could have altered sediment biogeochemistry by increasing evaporation. However, the presence of ammonium in the sediment is indicative of reduced soil conditions commonly observed in saturated soils (Pezeshki & DeLaune, 2012), suggesting that our manipulations did not lead to greater desiccation of sub‐surface sediments. Competition‐mediated suppression of cordgrass stem density may increase ammonium availability in sub‐surface sediments because of cordgrass's affinity for ammonium (Mozdzer et al., 2011). Increased ammonium availability at salt marsh transitional zones, where cordgrass stem density is restricted by pickleweed, may have important consequences for salt marsh structure and function. For example, elevated ammonium can increase the abundance of denitrifying microbes in marsh sediments and enhance N_2_O emissions (Avrahami et al., 2002).

In our study, we indirectly manipulated cordgrass stem density via plant–plant interactions. However, other factors can also affect the stem density of cordgrass and thus, may facilitate similar density‐dependent effects of cordgrass stem density on sub‐surface ammonium concentrations. For example, burrowing crabs increased cordgrass stem density at Kendall‐Frost Marsh, which corresponded with lower sub‐surface ammonium concentrations (Walker et al., 2020). This suggests that any environmental factor that alters the density of cordgrass stems could have indirect effects on ammonium concentrations in sub‐surface sediments.

Our finding that cordgrass stem density mediates sediment ammonium concentrations at mid‐marsh transitional zones is important considering the effects of anthropogenic climate change on tidal marsh plant communities. For instance, sea‐level rise is expected to affect inundation and salinity, and thereby influence plant distributions in Mediterranean salt marsh communities (Pennings & Callaway, 1992; Zedler, 1982). Shifts in plant distributions due to sea‐level rise may also influence the direction and intensity of plant–plant interactions. In fact, simulated sea‐level rise intensified the competitive effects of pickleweed (*S*. *pacifica*) on subordinate plant species, suggesting that sea‐level rise may intensify competitive interactions amongst salt marsh plants (Noto & Shurin, 2017). Such increased competitive abilities of pickleweed could further suppress cordgrass populations and alter sediment biogeochemistry, which may have reverberating effects on important salt marsh functions including nitrogen removal and carbon sequestration. However, we should note that the impacts of sea‐level rise on cordgrass may not always be negative—accelerated sea‐level rise was associated with lower marsh cordgrass (*S*. *alterniflora*) displacing higher‐marsh species in New England salt marshes (Donnelly & Bertness, 2001).

Despite considerable spatial and temporal variation, our study uncovered links between the population‐level consequences of interspecific interactions and local ecosystem function—suggesting that biotic interactions help mediate patterns of salt marsh ecosystem function. Our discovery demonstrated that competition between plant species can influence soil chemistry. Our study highlights the need to further understand the mechanisms by which cordgrass affects local sediment biogeochemistry, and how these effects are impacted by interactions with neighboring plants and under projected sea‐level rise scenarios.

## CONFLICT OF INTEREST

The authors do not have any conflicts of interest.

## AUTHOR CONTRIBUTIONS

**Janet B. Walker:** Conceptualization (equal); Data curation (equal); Formal analysis (supporting); Investigation (equal); Methodology (equal); Supervision (equal); Validation (equal); Visualization (equal); Writing – original draft (equal); Writing – review & editing (equal). **Shelby Rinehart:** Conceptualization (equal); Data curation (equal); Investigation (equal); Methodology (equal); Validation (equal); Visualization (equal); Writing – original draft (equal); Writing – review & editing (equal). **Gabriel Greenberg‐Pines:** Data curation (equal); Formal analysis (lead); Investigation (equal); Methodology (equal); Validation (equal); Visualization (equal); Writing – original draft (equal); Writing – review & editing (equal). **Wendi K. White:** Data curation (supporting); Investigation (equal); Methodology (equal); Validation (equal); Visualization (equal); Writing – review & editing (equal). **Ric DeSantiago:** Data curation (supporting); Investigation (equal); Methodology (equal); Validation (equal); Visualization (equal); Writing – review & editing (equal). **David A. Lipson:** Conceptualization (equal); Data curation (equal); Funding acquisition (equal); Investigation (equal); Project administration (equal); Resources (equal); Supervision (equal); Validation (equal); Writing – review & editing (equal). **Jeremy D. Long:** Conceptualization (equal); Data curation (equal); Funding acquisition (equal); Investigation (equal); Methodology (equal); Project administration (equal); Resources (equal); Supervision (equal); Validation (equal); Visualization (equal); Writing – original draft (equal); Writing – review & editing (equal).

### OPEN RESEARCH BADGES

This article has been awarded Open Materials, Open Data Badges. All materials and data are publicly accessible via the Open Science Framework at https://doi.org/10.5281/zenodo.5998548.

## Supporting information

Supplementary Material

## Data Availability

The data that support the findings of this study are openly available in Janet Walker's GitHub repository at https://github.com/janwalker, Repository name: AG‐Comp‐Sediment‐Biogeochem. Data is published with a DOI via Zenodo ‐ 10.5281/zenodo.5998548 (https://doi.org/10.5281/zenodo.5998548).
