# Supplementary material for: Aboveground competition influences density‐dependent effects of cordgrass on sediment biogeochemistry
Source: Ecol Evol. 2022 Mar 22;12(3):e8722. doi: 10.1002/ece3.8722 (PMC8939245; doi:10.1002/ece3.8722)
Supplement: Supplementary file 1 — Supplementary Material [file ECE3-12-e8722-s001.docx]

**Supplemental 1**

Walker, J.B., S. Rinehart, G. Greenberg-Pine, W.K. White, R. DeSantiago, D.A. Lipson, and J.D. Long. Aboveground competition influences density-dependent effects of cordgrass on sediment biogeochemistry. Ecology and Evolution.

**Table S1. Replication of treatment plots, porewater sippers, and sediment cores.** Plot replication differed between sites and years because of logistical limitations. At KF1, we lost three Pickleweed Removal plots and three Mixed plots due to erosion and stem borer impact. In 2017, we were able to increase plot replication because we only had one site (SDL2). Porewater sipper and sediment core replication were limited due to budget constraints.

|  |  | **Sample Size** | | |
| --- | --- | --- | --- | --- |
|  |  | **KF1** | **SDL1** | **SDL2** |
| **Treatment plots** | Pickleweed Removal | 5 | 8 | 10 |
|  | Mixed | 13 | 16 | 20 |
|  | Cordgrass Removal | 7 | 8 | 10 |
|  |  |  |  |  |
| **Porewater sippers (salinity, DOC, nitrate, ammonium)** | Pickleweed Removal | 5 | 8 | 7 |
|  | Mixed | 13 | 16 | 16 |
|  | Cordgrass Removal | 7 | 8 | 8 |
|  |  |  |  |  |
| **Sediment cores (iron oxide)** | Pickleweed Removal | - | - | 4 |
|  | Mixed | - | - | 8 |
|  | Cordgrass Removal | - | - | 4 |

**Table S2. Output table of models of above- and belowground biomass.** Results of the GLMM with a zero-inflation parameter (Gaussian) testing the effect of treatment (Cordgrass Removal, Pickleweed Removal, Mixed) on aboveground biomass and the linear model testing the effect of treatment on belowground biomass.

|  | **Dependent Variables** | | | | | |
| --- | --- | --- | --- | --- | --- | --- |
|  | **Aboveground biomass** | | | **Belowground biomass** | | |
|  | *df* | χ^2^ | *p* | *df* | *F* | *p* |
| **Treatment** | 2 | 1.104 | 0.576 | 2 | 1.137 | 0.335 |
| **Plant** | 1 | 3.191 | 0.074 | 1 | 42.268 | <0.001 |
| **Initial cordgrass stem density** | 1 | 2.326 | 0.127 | 1 | 0.0721 | 0.403 |
| **Treatment x Plant** | 2 | 0.0002 | 0.999 | 2 | 1.215 | 0.311 |
| **Zero-inflation term** | | | <0.001 |  |  |  |

**Table S3. Output table of linear models comparing treatment effects on biogeochemistry.** Sites were pooled for ammonium and then again for nitrate; however, models were run separately for each site for salinity and DOC due to bimodal distributions.

|  |  | Dependent Variables | | | | | | | | | |
| --- | --- | --- | --- | --- | --- | --- | --- | --- | --- | --- | --- |
|  |  | **Salinity**  **(ppt)** | | **DOC**  **(UV, A260nm)** | |  |  | **Nitrate (uM)** | | **Ammonium (uM)** | |
| **Site** | *df* | χ^2^ | p | χ^2^ | p |  | *df* | χ^2^ | p | χ^2^ | p |
| **KF1** |  |  |  |  |  | Treatment | *2* | 10.718 | 0.00471 | 24.976 | <0.001 |
| Treatment | 2 | 2.153 | 0.341 | 3.574 | 0.167 | Initial cordgrass stem density | 1 | 0.054 | 0.816 | 1.333 | 0.248 |
| Initial cordgrass stem density | 1 | 1.758 | 0.185 | 0.652 | 0.419 |  |  |  |  |  |  |
| **SDL1** |  |  |  |  |  |  | | | | | |
| Treatment | 2 | 0.158 | 0.924 | 2.911 | 0.234 |  |  |  |  |  |  |
| Initial cordgrass stem density | 1 | 0.176 | 0.675 | 1.039 | 0.308 |  |  |  |  |  |  |
| **SDL2** |  |  |  |  |  |  |  |  |  |  |  |
| Treatment | 2 | 4.681 | 0.096 | 3.097 | 0.213 |  |  |  |  |  |  |
| Initial cordgrass stem density | 1 | 0.344 | 0.557 | 0.367 | 0.545 |  |  |  |  |  |  |

**Fig. S1** Initial cordgrass stem density in treatment plots across sites. Lines inside boxes are median values, box limits are Q1 and Q3, and whiskers represent non-outlier ranges.

**Fig. S2** Cordgrass stem height in treatment plots pooled across site. Zero values in Cordgrass Removal plots demonstrate an effective manipulation. Lines inside boxes are median values, box limits are Q1 and Q3, and whiskers represent non-outlier ranges. Point shape represents site.

**Fig. S3** Belowground biomass in treatment plots at SDL2 for each plant species – cordgrass and pickleweed. Lines inside boxes are median values, box limits are Q1 and Q3, and whiskers represent non-outlier ranges. Letters represent significant differences between plants (Tukey HSD test; α = 0.05).

**Fig. S4** Pickleweed canopy height in treatment plots pooled across site. Zero values in Pickleweed Removal plots demonstrate an effective manipulation. Lines inside boxes are median values, box limits are Q1 and Q3, and whiskers represent non-outlier ranges. Letters represent significant differences between treatments (Tukey HSD test; α = 0.05). Point shapes represent sites.

**Fig. S5** Ammonium concentrations versus cordgrass stem density in mixed treatments only. Shapes represent site. Line represents a significant linear regression.

**Fig. S6** Porewater salinity in treatment plots by site. Lines inside boxes are median values, box limits are Q1 and Q3, and whiskers represent non-outlier ranges.

**Fig. S7** Porewater dissolved organic carbon, expressed as UV absorbance at A260 nm, in treatment plots by site. Lines inside boxes are median values, box limits are Q1 and Q3, and whiskers represent non-outlier ranges.

**Fig. S8** Porewater nitrate in treatment plots pooled across sites. Lines inside boxes are median values, box limits are Q1 and Q3, and whiskers represent non-outlier ranges. Letters represent significant differences between treatments (Tukey HSD test; α = 0.05). Point shapes represent sites.

**Fig. S9** (A) Proportional Fe (III), a proxy for sediment oxygenation, in treatment plots at depths of 0-10 cm and 10-20 cm at SDL2. Lines inside boxes are median values, box limits are Q1 and Q3, and whiskers represent non-outlier ranges. Letters represent a significant interaction between treatment and depth (Tukey HSD test; α = 0.05). (B) Proportional Fe (III) versus cordgrass stem density in shallow (0-10 cm) and deep sediments (10-20 cm) at SDL2. Point colors represent treatments. Line represents a significant linear regression across treatments at depth 10-20 cm. Proportional Fe (III) is calculated as Fe (III)/total soluble Fe.
